# Intimate Partner Violence, Mental Health Symptoms, and Modifiable Health Factors in Women During the COVID-19 Pandemic in the US

**DOI:** 10.1001/jamanetworkopen.2023.2977

**Published:** 2023-03-14

**Authors:** Arielle A. J. Scoglio, Yiwen Zhu, Rebecca B. Lawn, Audrey R. Murchland, Laura Sampson, Janet W. Rich-Edwards, Shaili C. Jha, Jae H. Kang, Karestan C. Koenen

**Affiliations:** 1 Department of Natural and Applied Sciences, Bentley University, Waltham, Massachusetts; 2 Department of Epidemiology, Harvard T.H. Chan School of Public Health, Boston, Massachusetts; 3 Division of Women’s Health, Department of Medicine, Brigham and Women’s Hospital and Harvard Medical School, Boston, Massachusetts; 4 Channing Division of Network Medicine, Department of Medicine, Brigham and Women’s Hospital and Harvard Medical School, Boston, Massachusetts; 5 Department of Social and Behavioral Sciences, Harvard T.H. Chan School of Public Health, Boston, Massachusetts; 6 Psychiatric and Neurodevelopmental Genetics Unit, Department of Psychiatry, Massachusetts General Hospital, Boston

## Abstract

**Importance:**

During the COVID-19 pandemic, the prevalence and severity of intimate partner violence (IPV) increased. Associations between IPV and mental health symptoms and modifiable health factors early in the pandemic have yet to be explored.

**Objective:**

To prospectively investigate the association of IPV with greater risk of mental health symptoms and adverse health factors during the COVID-19 pandemic in 3 cohorts of female participants.

**Design, Setting, and Participants:**

This cohort study used observational data from 3 prospective, population-based, longitudinal cohorts in the US: the Nurses’ Health Study II, Growing Up Today Study, and Nurses’ Health Study 3. Data analyzed included baseline and follow-up survey responses about IPV experiences early in the pandemic (March-September 2020); mental health domains of depression, anxiety, and posttraumatic stress symptoms (PTSS); and modifiable health factors (May 2020-October 2021). Female participants (both health care professionals and non–health care workers) aged 21 to 60 years from the 3 cohorts were included in the full analytic sample.

**Exposures:**

Experience of IPV measured by the Relationship Assessment Tool and fear of partner.

**Main Outcomes and Measures:**

Mental health symptoms, including depression, anxiety, and PTSS, and modifiable health factors, including sleep duration, sleep quality, physical activity, alcohol use, and use of alcohol or other substances to cope with stress.

**Results:**

The full analytic sample included 13 597 female participants with a mean (SD) age of 44 (10.6) years. Accounting for sociodemographic factors and prepandemic mental health symptoms and correcting for multiple testing, experiencing IPV was associated with higher endorsement of depression (odds ratio [OR], 1.44; 95% CI, 1.38-1.50), anxiety (OR, 1.31; 95% CI, 1.26-1.36), and PTSS (OR, 1.22; 95% CI, 1.15-1.29) in random-effects meta-analyses across the 3 cohorts. The IPV experience was also associated with poorer sleep quality (OR, 1.21; 95% CI, 1.16-1.26), shorter sleep duration (OR, 1.13; 95% CI, 1.08-1.19), increased use of alcohol (OR, 1.10; 95% CI, 1.06-1.14), and use of alcohol or other substances to cope with stress (OR, 1.13; 95% CI, 1.08-1.18) across all cohorts as well as decreased physical activity (OR, 1.17; 95% CI, 1.09-1.26) in the Nurses’ Health Study II only.

**Conclusions and Relevance:**

Results of the study showed that IPV experiences at the start of the pandemic were associated with worse mental health symptoms and modifiable health factors for female participants younger than 60 years. Screening and interventions for IPV and related health factors are needed to prevent severe, long-term health consequences.

## Key Points

**Question:**

Was intimate partner violence (IPV) early in the COVID-19 pandemic in the US associated with adverse mental health symptoms and modifiable health factors in women?

**Findings:**

In this cohort study of 3 nationwide cohorts involving 13 597 female participants, experiencing IPV was associated with higher endorsement of mental health symptoms, shorter sleep duration, poorer sleep quality, and increased use of alcohol or other substances.

**Meaning:**

The findings of this study suggest that IPV during the first 1.5 years of the pandemic in the US was associated with harmful health consequences; screening and interventions for IPV and related health factors are needed to prevent such outcomes.

## Teaser

This cohort study evaluates the risk of depression, anxiety, and posttraumatic stress symptoms and adverse health outcomes among female adults in potentially violent relationships in the early days of pandemic-related restrictions.

## Introduction

Intimate partner violence (IPV) is defined as physical, sexual, or psychological harm by a current or former partner.^1^ Women experience a substantial burden of IPV: approximately one-third of women who have been in a relationship have experienced physical or sexual abuse.^2,3,4^ Early in the COVID-19 pandemic, IPV experts expressed concern that COVID-19 mitigation actions meant to ensure public safety, such as stay-at-home orders, might further isolate individuals in abusive relationships and increase the prevalence and severity of violence.^5,6,7,8^ The pandemic played a role in the exacerbated burden of external stressors for many households, such as financial hardship, job loss, and food or housing insecurity.^4,9,10,11^ Increased isolation of women in potentially violent living situations and external stressors on their partners may be associated with heightened risk of IPV exposure.^8^ As expected, IPV increased globally in 2020.^12^ In the US, calls to domestic violence hotlines^13,14^ increased in the first months of the pandemic.

Outside of the pandemic context, IPV is a risk factor for poor health outcomes, including depression and cardiometabolic diseases.^15,16,17,18^ A potential pathway to adverse physical health outcomes may be changes in modifiable health factors, such as sleep, substance use, or exercise.^19,20^ It is not yet known how IPV during the pandemic may affect modifiable health factors. A limited number of studies used a cross-sectional^21,22,23,24^ or qualitative design^25^ to examine the association between IPV experience and mental health and behavioral outcomes during the first year of the pandemic. These prior studies have found that IPV was associated with mental health problems, such as depression and anxiety symptoms, and with adverse health factors, such as increased substance use and COVID-19 exposure risk-taking (eg, gathering indoors with people not belonging to one’s household during a lockdown).^22,23,26^ However, to our knowledge, no prospective studies have examined the associations of IPV with health factors and mental health symptoms in large, population-based cohorts. Intimate partner violence affects health across the life course,^27^ and the implications of IPV may manifest differently for individuals with IPV history. Therefore, a prospective design with information about prepandemic health and IPV history is instrumental in contextualizing the implications of IPV during the pandemic.

Herein, we aimed to extend previous studies by prospectively investigating the association of IPV with greater risk of mental health symptoms and adverse health factors during the COVID-19 pandemic in 3 cohort samples of female participants aged 21 to 60 years. We hypothesized that individuals who experienced IPV early in the pandemic would be at higher risk for mental health problems and adverse modifiable health factors, when accounting for prior depression and anxiety. Furthermore, we explored whether the role of IPV in these outcomes differed by prior experience of IPV.

## Methods

### Study Design and Population

This cohort study consisted of participants from 3 prospective cohorts: the Nurses’ Health Study II (NHS II), Growing Up Today Study (GUTS), and Nurses’ Health Study 3 (NHS3).^28^ The details on sampling and recruitment are provided in the eMethods in Supplement 1. The Brigham and Women's Hospital and the Harvard T.H. Chan School of Public Health Institutional Review Boards approved the study protocol. Completion of the questionnaires by participants was considered to be implied consent. We followed the Strengthening the Reporting of Observational Studies in Epidemiology (STROBE) reporting guideline.^29^

From April to May 2020, participants who had completed the most recent cohort questionnaire were invited to complete an online COVID-19 survey designed to examine experiences of both health care professionals (ie, those reporting to work in person at patient care institutions) and other individuals during the pandemic. After this initial survey, participants completed 3 monthly and 3 quarterly administered surveys, with the last quarterly questionnaire completed between March and October 2021.^30^ The month-1 questionnaire, which was mailed 28 days after the baseline questionnaire (May-June 2020), included an assessment of IPV for individuals who reported being in a relationship since March 2020 and were younger than 60 years. The age restriction was implemented because investigators were not able to follow mandatory state reporting guidelines for elder abuse. Month-1 survey returns were accepted through September 2020. While questionnaire return time varied among participants, there was no overlap in exposure and outcome assessment periods at the individual level. The minimum duration between exposure and outcome reporting was 1 month.

### Measures

Details about assessment contents and timing are included in the eMethods, eFigure 1, and eTable 1 in Supplement 1. We assessed experiences with IPV, mental health symptoms, modifiable health factors, covariates, and history of IPV.

We considered 2 exposures of IPV experiences, which were measured in the month-1 survey for each participant. First, participants who reported having a spouse, partner, or significant other since March 1, 2020, completed an adapted 6-item version of the Relationship Assessment Tool (RAT,^31^ formerly known as the Women’s Experience with Battering Scale), which measures emotional distress in response to IPV and has been found to have excellent internal consistency and validity. We used a standardized continuous summary score of RAT that ranged from 0 to 36 before standardization, with the highest score indicating the most severe level of emotional distress in response to IPV.^15,20^ In this sample, the quartile ranges of the RAT score were 0 to 1 for no distress (first quartile), 1 to 2 for very low distress (second quartile), 2 to 6 for mild distress (third quartile), and 6 to 36 for moderate to high distress (fourth quartile) in response to IPV. Second, we included in this survey a single item to assess fear of partner (ie, yes or no to the question, *Since March 1, 2020, have you ever felt afraid of your spouse/partner/significant other?*).^32^ Associations between these 2 exposure measures (RAT score and fear of partner) and health were assessed separately in subsequent analyses.

We assessed the following domains of mental health: depression, anxiety, and posttraumatic stress symptoms (PTSS), which are common responses to experiencing IPV.^33^ Current depression and anxiety were assessed with the 4-item Patient Health Questionnaire.^34^ The time frame of inquiry was adapted to cover the past 7 days. Current PTSS were assessed using the 6-item Impact of Events Scale,^35^ which was adapted to reflect the current context (eg, *I thought about COVID-19 or other current events when I didn’t mean to* and *I was aware that I had a lot of feelings about COVID-19 or other current events, but I didn’t deal with them*). To minimize missing data due to loss to follow-up, we considered the highest level of distress over follow-up (1-17 months after IPV assessment) for each domain separately.

Specific modifiable health factors that we examined were reported sleep quality, sleep duration, physical activity, alcohol use, and use of alcohol or other substances to cope with stress in 2020 to 2021. These factors were selected because of their known associations with IPV and long-term physical health^36,37^ outside of the pandemic context.

We adjusted for sociodemographic factors, including age at the baseline survey, race and ethnicity (coded as White [given that >95% of the samples self-identified as non-Hispanic White] or Other [American Indian or Alaska Native, Asian, Black or African American, Hispanic, Native Hawaiian or Other Pacific Islander, and multiracial]; race and ethnicity were assessed to identify possible disparities, with acknowledgment that race is socially constructed and its measurement can be a proxy for racism^38^), educational level as a proxy for socioeconomic status (whose information was used was based on data availability: the partner’s was used in NHS II and NHS3, whereas the participant’s was used in GUTS), active health care professional status during the COVID-19 pandemic (yes or no), partnership status (eg, married, separated or divorced, widowed, or single; data from the most recent biennial or modular surveys before 2020 were used), sexual orientation (heterosexual or not heterosexual), and depression or anxiety prior to exposure ascertainment (eMethods in Supplement 1). For all covariates with missing data, missing indicators were used.

We were able to examine history of IPV in 2 of the 3 cohorts. Exposure to IPV was measured in 2001 and 2008 for NHS II and in 2007 for GUTS (eMethods in Supplement 1).

### Statistical Analysis

All analyses were conducted in R, version 4.1.0 (R Core Team), and 2-sided tests were used. Multiple testing burden was accounted for using a false discovery rate correction. The statistical significance threshold was corrected *P* < .05.

Descriptive analyses were performed to characterize the distributions of sociodemographic and health characteristics by IPV exposure in each cohort. Prevalence of IPV, as indicated by the distribution of the continuous RAT score and endorsement of individual items, was also examined. To assess the associations between IPV and each outcome measure, we fitted 16 logistic regression models in each cohort separately (2 exposures [RAT score and fear of partner] and 8 outcomes), resulting in a total of 48 models. After the stratified analysis within each cohort, a random-effects meta-analysis was performed per outcome to summarize the association with IPV across the 3 cohorts. Inverse probability weighting was implemented to address loss to follow-up (eMethods in Supplement 1).

In a secondary analysis, to evaluate whether patterns of associations between IPV during the pandemic and health outcomes were different among individuals with vs without prior IPV exposure, we repeated the primary analyses and stratified by IPV history in NHS II and GUTS, for which data on prior IPV exposure were available. All main and secondary analyses were adjusted for the covariates.

## Results

### Descriptive Analysis

The final analytic sample included 13 597 female participants (3503 from NHS II, 2858 from GUTS, and 7236 from NHS3) with a mean (SD) age of 44 (10.6) years. Most participants identified as being of non-Hispanic White race (96.3%) and being heterosexual (92.8%). Participants of the NHS II, GUTS, and NHS3 had a mean (SD) age of 58 (1.4) years, 33 (3.3) years, and 42 (7.5) years, respectively. At baseline in spring 2020, active health care professionals composed 21.2% of the GUTS, 57.4% of the NHS II, and 76.0% of the NHS3 participants. In the full sample, the proportions of active health care professionals were comparable across 4 quartiles of the RAT score (Table). Prevalence of anxiety and PTSS was high in the samples, with 37.9% of participants in NHS II, 61.9% in GUTS, and 50.5% in NHS3 reporting anxiety and 38.4% in NHS II, 56.7% in GUTS, and 51.3% in NHS3 reporting PTSS at 1 or more of the follow-up interviews. Depressive symptoms were reported by 21.2% of the NHS II, 37.4% of the GUTS, and 32.1% of the NHS3 participants.

**Table.** Sociodemographic and Health Characteristics in the Analytic Samples (N = 13 597)

Characteristic	No. of participants (%) in each RAT score quartile			
	First (mean score = 0.2)	Second (mean score = 1.5)	Third (mean score = 3.7)	Fourth (mean score = 10.7)
No. of participants	3400	3399	3399	3399
Fear of partner^a^	19 (0.6)	19 (0.6)	18 (0.5)	207 (6.1)
Amount of time spent with partner due to COVID-19 pandemic restrictions^a^				
Decreased	202 (5.9)	223 (6.6)	194 (5.7)	323 (9.5)
No change	923 (27.2)	834 (24.6)	875 (25.8)	797 (23.4)
Increased	2258 (66.4)	2327 (68.6)	2317 (68.2)	2259 (66.5)
NA or missing data	16 (0.5)	10 (0.3)	12 (0.4)	20 (0.6)
Change in relationship quality				
Worsened	77 (2.3)	145 (4.3)	280 (8.3)	780 (23.0)
No change	2252 (66.3)	2262 (66.6)	2179 (64.2)	1918 (56.5)
Improved	1058 (31.1)	987 (29.1)	928 (27.4)	685 (20.2)
NA or missing data	11 (0.3)	3 (0.1)	6 (0.2)	14 (0.4)
Living arrangement at baseline				
Alone	104 (3.1)	96 (2.8)	86 (2.5)	107 (3.1)
With partner^a^	3147 (92.6)	3170 (93.3)	3158 (92.9)	3149 (92.6)
With children	46 (1.4)	47 (1.4)	39 (1.1)	53 (1.6)
With others^b^	95 (2.8)	79 (2.3)	108 (3.2)	85 (2.5)
With pets	8 (0.2)	6 (0.2)	8 (0.2)	5 (0.1)
Age, mean (SD), y	46.56 (11.16)	42.58 (9.76)	43.42 (10.94)	45.09 (10.11)
Active health care professional status during the COVID-19 pandemic	2046 (60.2)	2137 (62.9)	1888 (55.5)	2043 (60.1)
Non-Hispanic White race	3284 (96.6)	3271 (96.2)	3278 (96.4)	3266 (96.1)
Other race and ethnicity^c^	116 (3.4)	128 (3.8)	121 (3.6)	133 (3.9)
Partnership status				
Married	2375 (69.9)	2238 (65.8)	2287 (67.3)	2428 (71.4)
Divorced	143 (4.2)	129 (3.8)	109 (3.2)	138 (4.1)
Widowed	16 (0.5)	5 (0.1)	4 (0.1)	6 (0.2)
Domestic partnership	140 (4.1)	164 (4.8)	201 (5.9)	174 (5.1)
Separated	17 (0.5)	16 (0.5)	28 (0.8)	32 (0.9)
Never married or in a relationship	622 (18.3)	799 (23.5)	688 (20.2)	547 (16.1)
Missing data	87 (2.6)	48 (1.4)	82 (2.4)	74 (2.2)
Spouse, partner, or participant educational level				
≤High school diploma	261 (7.7)	262 (7.7)	280 (8.2)	311 (9.1)
College degree	1668 (49.1)	1586 (46.7)	1647 (48.5)	1696 (49.9)
Graduate school	805 (23.7)	724 (21.3)	813 (23.9)	755 (22.2)
NA or missing data	666 (19.6)	827 (24.3)	659 (19.4)	637 (18.7)
Sexual orientation				
Heterosexual	3167 (93.1)	3131 (92.1)	3201 (94.2)	3120 (91.8)
Not heterosexual	124 (3.6)	127 (3.7)	112 (3.3)	122 (3.6)
Missing data	109 (3.2)	141 (4.1)	86 (2.5)	157 (4.6)
Depression or anxiety prior to exposure ascertainment				
No	2729 (80.3)	2498 (73.5)	2530 (74.4)	2261 (66.5)
Yes	671 (19.7)	899 (26.4)	865 (25.4)	1136 (33.4)
Missing data	0 (0.0)	2 (0.1)	4 (0.1)	2 (0.1)
Depression	709 (21.7)	848 (26.0)	1023 (31.1)	1391 (42.8)
Anxiety	1314 (40.1)	1550 (47.5)	1699 (51.6)	1919 (59.1)
PTSS	1356 (41.4)	1518 (46.6)	1677 (51.0)	1855 (57.2)
Decreased physical activity	1068 (35.2)	1168 (39.1)	1221 (40.6)	1239 (42.0)
Poorer sleep quality	902 (29.7)	1058 (35.5)	1192 (39.6)	1289 (43.6)
Decreased sleep duration	876 (28.9)	1038 (34.8)	1065 (35.4)	1156 (39.1)
Increased alcohol use	877 (27.3)	992 (30.9)	1053 (32.5)	1099 (34.5)
Use of alcohol or other substances to cope	612 (23.6)	693 (27.5)	790 (31.0)	809 (33.1)

Abbreviations: NA, not applicable; PTSS, posttraumatic stress symptoms; RAT, Relationship Assessment Tool.

^a^ Partner refers to spouse, domestic partner, or significant other.

^b^ Others in a living arrangement included individuals living with adult family members or non–family members.

^c^ Other race and ethnicity included American Indian or Alaska Native, Asian, Black or African American, Hispanic, Native Hawaiian or Other Pacific Islander, and multiracial.

The Table summarizes the sociodemographic and health characteristics of participants in the full analytic sample. An association between the RAT score and fear of partner was observed: fear of partner was reported by a much higher percentage of participants in the highest RAT score quartile (fourth) than in the first 3 quartiles (6.1% vs 0.6%, 0.6%, and 0.5%). We summarized the IPV item endorsements in the analytic samples across the 3 cohorts in eFigure 2 in Supplement 1.

### IPV and Mental Health Symptoms

**Figure 1.**
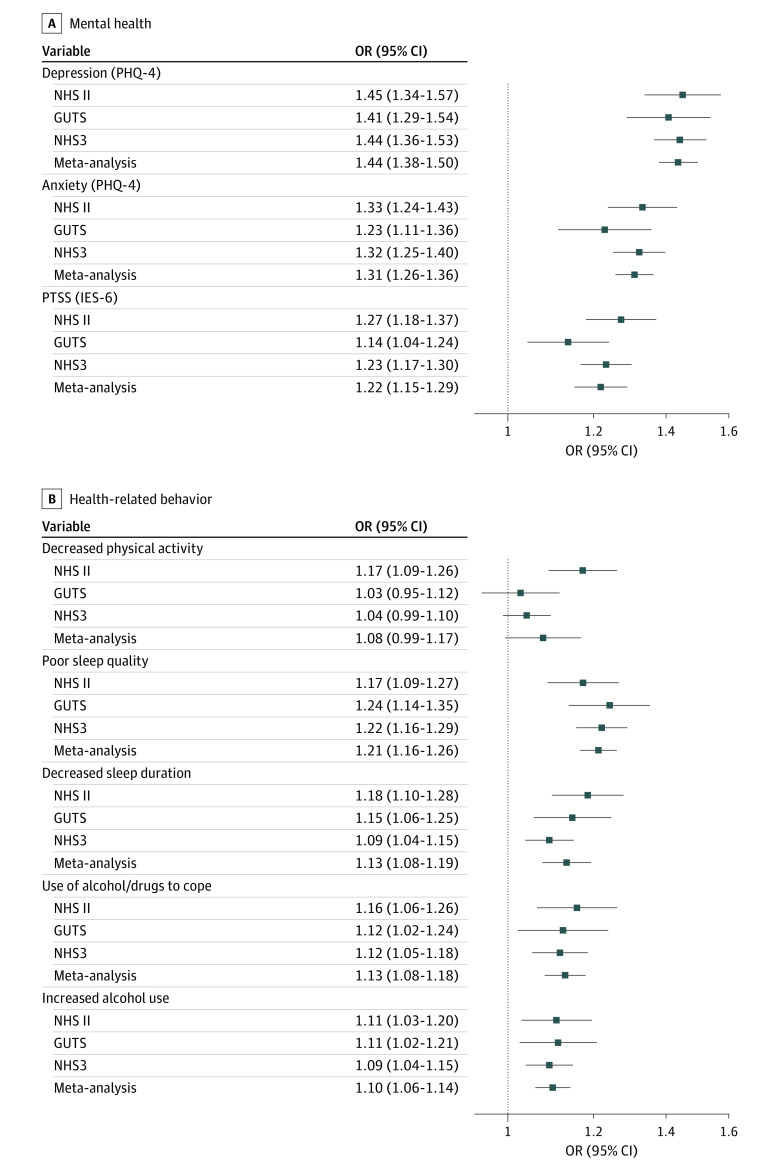
Associations of the Relationship Assessment Tool Score at Month 1 With Mental Health and Health-Related Behavior During Follow-up Estimates were obtained with random-effects meta-analysis across the 3 cohorts. All models were adjusted for age at baseline, race and ethnicity, educational level as a proxy for socioeconomic status, health care professional status, partnership status, sexual orientation, and depression or anxiety prior to exposure ascertainment. GUTS indicates Growing Up Today Study; IES-6, 6-item Impact of Events Scale; NHS II, Nurses’ Health Study II; NHS3, Nurses’ Health Study 3; OR, odds ratio; PHQ-4, 4-item Patient Health Questionnaire; PTSS, posttraumatic stress symptoms.

Accounting for depression and anxiety before exposure ascertainment, we found in meta-analyses across all 3 cohorts that experiencing IPV was associated with depression, anxiety, and PTSS during follow-up. Experiencing IPV at month 1 was associated with higher odds of depression (odds ratio [OR], 1.44; 95% CI, 1.38-1.50), anxiety (OR, 1.31; 95% CI, 1.26-1.36), and PTSS (OR, 1.22; 95% CI, 1.15-1.29) during follow-up (Figure 1; eTable 2 in Supplement 1). Cohort-specific estimates were comparable.

**Figure 2.**
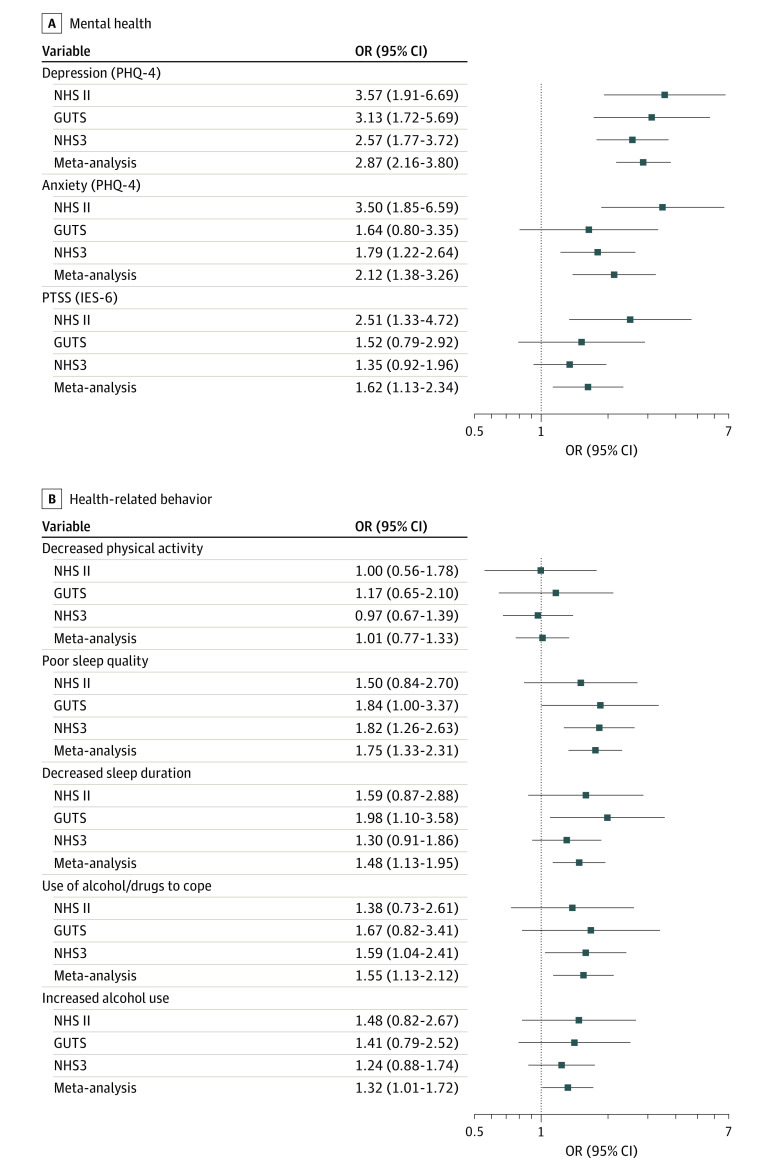
Associations Between Reported Fear of Spouse, Partner, or Significant Other at Month 1 and Mental Health and Health-Related Behavior During Follow-up Estimates were obtained with random-effects meta-analysis across the 3 cohorts. All models were adjusted for age at baseline, race and ethnicity, educational level as a proxy for socioeconomic status, health care professional status, partnership status, sexual orientation, and depression or anxiety prior to exposure ascertainment. GUTS indicates Growing Up Today Study; IES-6, 6-item Impact of Events Scale; NHS II, Nurses’ Health Study II; NHS3, Nurses’ Health Study 3; OR, odds ratio; PHQ-4, 4-item Patient Health Questionnaire; PTSS, posttraumatic stress symptoms.

Fear of one’s partner at month 1 was associated with higher odds of depression (OR, 2.87; 95% CI, 2.16-3.80), anxiety (OR, 2.12; 95% CI, 1.38-3.26), and PTSS (OR, 1.62; 95% CI, 1.13-2.34) during follow-up in a meta-analysis across cohorts (Figure 2; eTable 3 in Supplement 1). There were some differences between cohorts. Feeling afraid was not associated with PTSS in the NHS3 cohort. In the GUTS cohort, fear of one’s partner was not associated with anxiety or PTSS. The 95% CIs for associations between fear of partner and mental health symptoms were relatively wide due to the small numbers of individuals reporting this item.

### IPV and Modifiable Health Factors

Overall, the magnitude of association between IPV and modifiable health factors was smaller than that observed between IPV and mental health symptoms. In a meta-analysis across cohorts, we found that experiencing IPV was associated with poorer sleep quality (OR, 1.21; 95% CI, 1.16-1.26), decreased sleep duration (OR, 1.13; 95% CI, 1.08-1.19), increased alcohol use (OR, 1.10; 95% CI, 1.06-1.14), and use of alcohol or other substances to cope with stress (OR, 1.13; 95% CI, 1.08-1.18). In the NHS II cohort only, experiencing IPV was associated with decreased physical activity (OR, 1.17; 95% CI, 1.09-1.26) (Figure 1; eTable 2 in Supplement 1).

Fear of one’s partner was associated with decreased sleep duration (OR, 1.48; 95% CI, 1.13-1.95), poorer sleep quality (OR, 1.75; 95% CI, 1.33-2.31), increased alcohol use (OR, 1.32; 95% CI, 1.01-1.72), use of alcohol or other substances to cope with stress (OR, 1.55; 95% CI, 1.13-2.12), and decreased physical activity (OR, 1.01; 95% CI, 0.77-1.33) in a meta-analysis across cohorts. In cohort-specific estimates, there were differences. In the NHS3 cohort, fear of one’s partner was associated only with poorer sleep quality (OR, 1.82; 95% CI, 1.26-2.63) and use of alcohol or other substances to cope with stress (OR, 1.59; 95% CI, 1.04-2.41). In GUTS, fear was associated only with poorer sleep quality (OR, 1.84; 95% CI, 1.00-3.37) and decreased sleep duration (OR, 1.98; 95% CI, 1.10-3.58). In NHS II, fear was not associated with any modifiable health factor during follow-up (Figure 2; eTable 3 in Supplement 1). Results from minimally adjusted models (ie, only adjusting for age and race and ethnicity) were similar (eTables 4 and 5 in Supplement 1).

### Secondary Analyses

**Figure 3.**
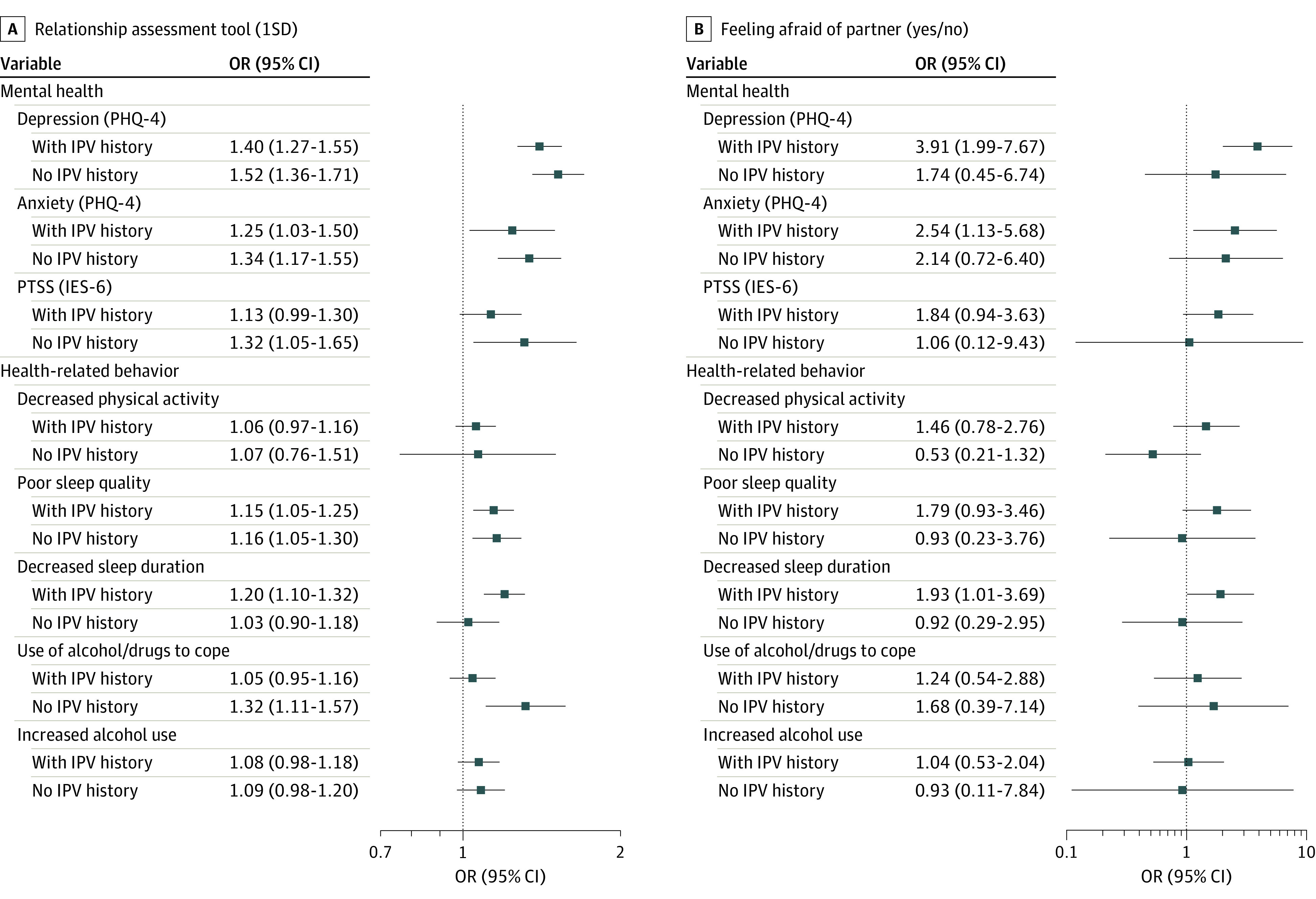
Stratified Analysis by Prior History of Intimate Partner Violence Estimates were obtained with random-effects meta-analysis across 2 cohorts. All models were adjusted for age at baseline, race and ethnicity, educational level as a proxy for socioeconomic status, health care professional status, partnership status, sexual orientation, and depression or anxiety prior to exposure ascertainment. IES-6 indicates 6-item Impact of Events Scale; IPV, intimate partner violence; OR, odds ratio; PHQ-4, 4-item Patient Health Questionnaire; PTSS, posttraumatic stress symptoms.

We conducted a stratified analysis by prior IPV history in the NHS II and GUTS cohorts. The prevalence of IPV history was 37.2% in NHS II and 40.2% in GUTS. Analyzing the cohorts together, we observed differences in associations with PTSS by IPV history, with ORs that were greater in magnitude among participants with no IPV history (OR, 1.32; 95% CI, 1.05-1.65) compared with individuals with IPV history before the pandemic (OR, 1.13; 95% CI, 0.99-1.30) (Figure 3). The association with use of alcohol or other substances to cope with stress was also greater in magnitude among participants with no IPV history (OR, 1.32; 95% CI, 1.11-1.57) compared with individuals with IPV history (OR, 1.05; 95% CI, 0.95-1.16) (Figure 3). The 95% CIs in these analyses were wide, indicating a lack of precision in estimates.

## Discussion

Overall, prevalence of depression in this study was comparable to the prevalence reported in other population-based assessments during the COVID-19 pandemic.^39,40^ However, anxiety and PTSS were more prevalent, which could be explained by higher distress among active health care professionals^41^ and the analytic strategy we used to capture the highest level of distress during follow-up. Participants in the full sample who experienced IPV had higher odds of experiencing depression, anxiety, and PTSS over the course of the first 1.5 years of the pandemic. We also found evidence of IPV’s association with modifiable health factors. Specifically, experience of IPV was associated with shorter sleep duration, poorer sleep quality, and increased use of alcohol or other substances in all 3 cohorts. Additionally, IPV was associated with decreased physical activity in the NHS II cohort. When examining the potential implications of IPV history, we found that participants with no IPV history who were exposed to IPV during the pandemic were at higher risk for PTSS and use of alcohol or other substances to cope with stress during the pandemic than participants who were not exposed to IPV. This finding may be attributed to the timing of IPV in that recent traumatic experiences may translate into current symptoms.^42,43^

The findings of this study have several key implications for both prevention and intervention. Intimate partner violence is a preventable crime associated with harmful health consequences. Routine and well-implemented screening^44,45^ for IPV, including follow-up and referrals for those receiving positive screening results,^46^ is critical during a time of increased risk (eg, COVID-19 pandemic). During the first years of the pandemic, access to safe housing and health care became more limited for those living in violent situations, as capacity in shelters and transitional housing decreased and many patients shifted to virtual health care appointments at home, from where it may not have been safe to seek help from clinicians.^46^ The modifiable health factors examined in the current study were implicated in substantial long-term health problems.^47,48,49^ Response and referral training to a broad spectrum of clinicians who may interface with women experiencing IPV may allow for quick interventions on the exposure and health risk factors,^50^ guarding against more severe health consequences in the future.

One unique aspect of this study was the assessment of IPV’s implications in the 3 cohorts of participants at different stages of their lives. While consistent patterns of associations emerged across the cohorts, some cohort-specific findings may indicate heightened vulnerability in older participants. Specifically, those who were enrolled in NHS II were more likely to report decreased physical activity after IPV exposure, but no association was identified in the 2 cohorts (GUTS and NHS3) with younger participants. Challenges in seeking support and accessing resources for older women who were in abusive relationships^51^ may have been exacerbated during the pandemic given the physical confinement at home. The trauma could also be compounded by elder abuse, which we were not able to assess. Promotion of physical activity among older women and increased public awareness and education about IPV are needed in times of collective stress, wherein support may come from formal and informal sources.^52^

### Strengths and Limitations

The current study had 3 main methodological strengths. First, we leveraged prospective data from over 13 000 female participants in 3 well-characterized longitudinal cohorts, with health assessments both before and during the COVID-19 pandemic. Second, the IPV measure we used focused on cognitive and emotional experiences of IPV, rather than episodic events. This approach aligns well with considerations about characterizing nuanced experiences of trauma during a period of heightened risk: individual responses to prolonged stress and terror may have implications for health beyond the frequency or incidence of violence.^53^ Third, by examining a range of health factors under a unified analytic framework, rather than focusing on a specific disorder, we provided a comprehensive overview of the outcome of IPV and found associations between domains of health, potentially minimizing the implications of biases present for any specific outcome and generating more relevant public health recommendations.^54^

Several limitations of this study also must be noted. First, to minimize loss to follow-up within the analytic sample and to capture severe stress response, we considered the highest distress level during follow-up for each domain separately. A future direction is therefore to examine patterns of change over a longer period of the pandemic, with repeatedly collected exposure and confounder information to further disentangle the association of IPV with mental health symptoms and modifiable health factors. Second, this study lacks representation of some groups given that the sample was composed of mostly White heterosexual women who were younger than 60 years, who had a higher socioeconomic status than the general population,^28^ and about whom we had limited information on IPV type or severity. Health outcomes of IPV may be exacerbated in individuals experiencing severe or certain types of violence, especially in the presence of other acute stressors, such as financial hardship and job insecurity, which we were unable to assess. We did not have information on the sex or gender identity of participants’ partners, and the study was underpowered to pursue any analysis examining effect modification by sexual orientation or by partner sex or gender identity. Additionally, IPV prevalence, severity, and implications for health are affected by racial and ethnic and socioeconomic differences due to health disparities and variations in help-seeking behaviors.^55,56^ Future work should explore IPV experiences and health consequences during the pandemic among males, persons with nonbinary identity, diverse racial and ethnic groups, and individuals from less advantaged socioeconomic strata.

## Conclusions

This cohort study extended previous research by prospectively examining the association of IPV with worse modifiable health factors and mental health symptoms in 3 population-based US cohorts during the first 1.5 years of the COVID-19 pandemic, a unique time of collective stress. Accounting for differences in prepandemic health, the analyses provided insights into health outcomes for female participants younger than 60 years who experienced IPV early in the pandemic. Such IPV exposure had mental and physical health costs for these individuals. Screening and interventions for IPV and related health factors are needed to prevent severe, long-term health consequences.

## Notes

Supplement 1.
eMethods.
**eTable 1.** Summary of Mental Health and Health-Related Behavior Outcome Measures **eFigure 1.** Timeline of Administration of Study Measures **eTable 2.** Associations Between the Relationship Assessment Tool Score at Month One and Mental Health and Health-Related Behavior During the Pandemic **eTable 3.** Associations Between Reported Feeling Afraid of Spouse/Partner/Significant Other at Month One and Mental Health and Health-Related Behavior During the Pandemic **eTable 4.** Minimally Adjusted (Age and Race/Ethnicity Adjusted) Associations Between the Relationship Assessment Tool Score at Month One and Mental Health and Health-Related Behavior During the Pandemic **eTable 5.** Minimally Adjusted (Age and Race/Ethnicity Adjusted) Associations Between Reported Feeling Afraid of Spouse/Partner/Significant Other at Month One and Mental Health and Health-Related Behavior During the Pandemic **eFigure 2.** Prevalence of Intimate Partner Violence Assessment Items in the Analytic Samples Across Three Cohorts

Supplement 2.
Data Sharing Statement
