# Supplementary material for: Intimate Partner Violence, Mental Health Symptoms, and Modifiable Health Factors in Women During the COVID-19 Pandemic in the US
Source: JAMA Netw Open. 2023 Mar 14;6(3):e232977. doi: 10.1001/jamanetworkopen.2023.2977 (PMC10015312; doi:10.1001/jamanetworkopen.2023.2977)
Supplement: Supplement 1. — eMethods. eTable 1. Summary of Mental Health and Health-Related Behavior Outcome Measures eFigure 1. Timeline of Administration of Study Measures eTable 2. Associations Between the Relationship Assessment Tool Score at Month One and Mental Health and Health-Related Behavior During the Pandemic eTable 3. Associations Between Reported Feeling Afraid of Spouse/Partner/Significant Other at Month One and Mental Health and Health-Related Behavior During the Pandemic eTable 4. Minimally Adjusted (Age and Race/Ethnicity Adjusted) Associations Between the Relationship Assessment Tool Score at Month One and Mental Health and Health-Related Behavior During the Pandemic eTable 5. Minimally Adjusted (Age and Race/Ethnicity Adjusted) Associations Between Reported Feeling Afraid of Spouse/Partner/Significant Other at Month One and Mental Health and Health-Related Behavior During the Pandemic eFigure 2. Prevalence of Intimate Partner Violence Assessment Items in the Analytic Samples Across Three Cohorts [file jamanetwopen-e232977-s001.pdf]

Supplemental Online Content

Scoglio AAJ, Zhu Y, Lawn RB, et al. Intimate partner violence, mental health symptoms, and modifiable health factors in women during the COVID-19 pandemic in the US. *JAMA Netw Open*. 2023;6(3):e232977. doi:10.1001/jamanetworkopen.2023.2977

eMethods.

eTable 1. Summary of Mental Health and Health-Related Behavior Outcome Measures

eFigure 1. Timeline of Administration of Study Measures

eTable 2. Associations Between the Relationship Assessment Tool Score at Month One and Mental Health and Health-Related Behavior During the Pandemic

eTable 3. Associations Between Reported Feeling Afraid of Spouse/Partner/Significant Other at Month One and Mental Health and Health-Related Behavior During the Pandemic

eTable 4. Minimally Adjusted (Age and Race/Ethnicity Adjusted) Associations Between the Relationship Assessment Tool Score at Month One and Mental Health and Health-Related Behavior During the Pandemic

eTable 5. Minimally Adjusted (Age and Race/Ethnicity Adjusted) Associations Between Reported Feeling Afraid of Spouse/Partner/Significant Other at Month One and Mental Health and Health-Related Behavior During the Pandemic

eFigure 2. Prevalence of Intimate Partner Violence Assessment Items in the Analytic Samples Across Three Cohorts

This supplementary material has been provided by the authors to give readers additional information about their work.

© 2023 Scoglio AAJ et al. *JAMA Network Open.*

eMethods

Participant Sampling and Selection

NHS II is an ongoing cohort study of women, which enrolled 116,429 registered nurses aged 24-42 years residing in 14 US states in 1989. NHS II participants were identified from registration listings in states with large populations and where nursing boards provided information on registered nurses' gender and age. GUTS participants are offspring of NHS II participants, and between 9 and 17 years of age at recruitment in 1996 (N= 27,793). Participants for GUTS were identified through contacting active NHSII participants who reported having at least one child. NHS3 is a currently recruiting cohort established in 2010 and originally enrolled female registered nurses, licensed practical/vocational nurses or nursing students born on or after January 1, 1965, who were living in the US or Canada. The sampling and recruitment strategies for NHS3 involved convenience sampling, contacts with professional organizations, and social media campaigns. GUTS and NHS3 include a small portion of men; we focused on female participants for this study.

In April-May 2020, participants who had completed the most recent cohort questionnaire in 2019 were invited to complete an online COVID-19 survey designed to examine experiences of participants (both healthcare professionals and other individuals) during the pandemic. The COVID-19 substudy baseline response rate was 71% in NHSII, 35% in GUTS, and 40% in NHS3. Details on the selection of participants at baseline are described elsewhere[35]. Compared to the original cohort source population, the substudy participants were generally older, but similar in other characteristics.

In NHSII there were 39,137 women who completed the COVID-19 baseline questionnaire in May-August 2020 and 33,696 (86%) who completed a month one questionnaire. In GUTS, the baseline Covid-19 questionnaire was returned by 6,725 participants, with 4,841 (72%) participants also completing month one follow-up. In NHS3, a total of 12,175 participants completed the COVID-19 baseline questionnaire and then 8,760 (72%) completed the month one follow-up survey.

Details on Measurement Ascertainment

Previous Experience of IPV. We restricted the stratified analyses by IPV history to NHSII and GUTS. Although IPV is also measured in NHS3, most participants had not yet reached the questionnaire in which it was assessed at the time of the COVID-19 substudy.

In NHS II, we examined a measure of any exposure to IPV victimization during adulthood, which was retrospectively assessed in 2001 and 2008, in substudies that focused on stress and PTSD. The items included: (1) "Has your spouse/partner ever made you feel afraid?" (2) "Have you ever been emotionally abused by your spouse/significant other?" (3) "Have you ever been hit, slapped, kicked or otherwise physically hurt by a spouse or significant other?" (4) "Has your spouse/significant other ever forced you to have sexual activities?" Endorsement of any of the items at either point was coded as having IPV history. In GUTS, IPV victimization was assessed in 2007 and queried about lifetime experiences of emotional abuse, physical abuse and physical abuse by an intimate partner. Specific items included the following: (1) "Has your partner ever made you feel afraid?" (2) "Have you ever been emotionally abused (e.g. threatened, insulted, yelled at, degraded) by your partner?" (3) "Have you ever been hit, slapped, kicked or otherwise physically hurt by your partner?" (4) "Did your partner ever use threats, force or verbal pressure

© 2023 Scoglio AAJ et al. *JAMA Network Open.*

to do something sexual when you did not want to? We used an ever/ never variable to capture experience of any type of past IPV.

Educational Attainment. Partner educational attainment was included as a proxy for socioeconomic status in NHSII and NHS3, given that participants were all trained healthcare professionals at the time of cohort enrollment. Data on participant education were not available for most participants. In NHSII, partner or spouse's education attainment was assessed in 1999, and participants reported their current partner or spouse's highest level of education (levels: less than high school, high school, 2-year college, 4-year college, and graduate school). In GUTS, because the cohort was not restricted to registered health professionals, study participants' education backgrounds were relatively more heterogeneous. Therefore, the study only collected information on the participants' own education attainment in 2014 (levels: high school or lower, college, graduate school). In NHS3, the education attainment level of each participant's partner or spouse was assessed in the first module they completed (levels: less than high school, high school, 2-year college, 4-year college, and graduate school).

Prior Depression and Anxiety. For prior psychological depression or anxiety, we focused on measures from biennial or modular surveys closest to the start of the pandemic. In NHS II, data came from the 2017 biennial survey, with established measures for depression (Center for Epidemiologic Studies Depression Scale; CES-D-10, cut-off set to be 10 or above[41]) and anxiety (Generalized Anxiety Disorder-7; GAD-7, cut-off set to be 10 or higher[42]) separately. In GUTS and NHS3, because no measures of symptoms were available, self-reported clinical diagnoses of depression and anxiety were used to create a similar indicator variable for estimated prior psychological distress. Because the questionnaires were structured in a way that not having the condition was marked as NA, the difference between actually missing and not having a physician diagnosed condition was ambiguous. For participants who did not have complete data on a pre-pandemic measure of psychological distress (7.3% in NHS II, 0% in GUTS, and 64% in NHS3; the large, between-sample differences were due to how and when depression and anxiety were assessed in each cohort), baseline depression or anxiety from the COVID-19 survey (one month before the IPV exposure ascertainment) was used to approximate prior levels of psychological distress. Specifically, in NHS3, many COVID-19 substudy participants had not completed the modules containing depression or anxiety measures before 2020. Therefore in this cohort we had to largely rely on the COVID-19 substudy baseline assessments.

IPV Measurement. The RAT has been shown to have excellent internal consistency and validity. The six items were: (1) "Usually my partner is sensitive to my needs (reverse coded)." (2) "I feel ashamed of the things my partner does to me." (3) "I feel like my partner keeps me prisoner." (4) "My partner respects my interests and independence (reverse coded)." (5) "My partner can scare me without laying a hand on me." (6) "I feel like I am programmed to react a certain way to my partner." Participants rated their agreement with each statement on a 7-point scale (0=strongly disagree, 6=strongly agree).
We also included the single item measure: "Since March 1, 2020, have you ever felt afraid of your spouse/partner/significant other?". Women who responded yes (vs. no) were categorized as being afraid of their partner.

© 2023 Scoglio AAJ et al. *JAMA Network Open.*

Individual RAT items. Overall, less than 15% of the participants reported at least some agreement with the RAT items, with most of these participants reporting that their partner was not sensitive to their needs or that they felt programmed to react a certain way to their partner. The least endorsed RAT item was “I feel like my partner keeps me prisoner.” Under 3% of the samples reported being afraid of their partner or spouse.

Attrition and inverse probability weighting

To minimize loss to follow up, for each outcome, we considered the highest level of distress over the course of follow-up. Because of this strategy, the rate of attrition varied by outcome measure, such that outcomes with the least number of measures had the highest missingness. Among the 3503 NHSII participants that responded to the IPV questions, the missingness of mental health outcomes (depression, anxiety, and PTSS) was under 3%. The missingness of health-related behavior measures was under 8%, except for use of substances to cope with stress, which had 21.5% missing since the coping questions were only administered at one time point. Among the 2858 GUTS participants in our analytic sample, the missingness patterns were similar: approximately 4% of the participants did not have mental health measures, and under 12% did not have health related behavior outcomes reported, except for use of substances to cope with stress (28.1%). Among the 7236 NHS3 participants, the missing rate of mental health measures was about 5%, and the missing rate of health-related behavior measures was about 14%, with the exception of use of substances to cope with stress (26.7%). To address any potential selection bias and loss of efficiency due to differential loss to follow up, we calculated inverse probability weights for each outcome separately, as a function of the exposures and baseline covariates. All analyses were weighted to represent the distribution of the analytic samples at baseline.

© 2023 Scoglio AAJ et al. *JAMA Network Open.*

eTable 1. Summary of mental health and health-related behavior outcome measures.

	Instrument or questionnaire items	Outcome definition	Frequency of assessments	Average duration between exposure and outcome assessments†
Mental Health				
Depression	Two items from PHQ-4: "In the past 7 days, have you been feeling down, depressed, or hopeless?" "In the past 7 days, have you had little interest or pleasure in doing things?"	Coded as 1 if sum score of two items >=3 (Kroenke et al. ,2003)	M2, M3, Q1, Q2, FQ	68 days
Anxiety	Two items from PHQ-4: "In the past 7 days, have you been feeling nervous, anxious, or on edge?" "In the past 7 days, have you been unable to stop or control worrying?"	Coded as 1 if sum score of two items >=3 (Kroenke et al. ,2007)	M2, M3, Q1, Q2, FQ	72 days
PTSS	Impact of Event Scale – 6 items	Coded as 1 if sum score >= 10 (Thoresen et al., 2010)	M2, M3, Q1, Q2, FQ	117 days
Health related behaviors				
Sleep quality	"In the past 7 days, how would you rate your sleep quality overall?"	Coded as 1 if the response was fairly bad or bad	M2, Q1	50 days
Sleep duration	"Has the amount of sleep you get changed in the past 30 days?"	Coded as 1 if any decrease was reported	M2, Q1	51 days
Physical activity	"Has the amount of physical activity you are doing changed in the past 30 days (include recreational and occupational activity)?"	Coded as 1 if any decrease was reported	M2, Q1	56 days
Alcohol use	"Has your consumption of the following changed, "Alcohol: beer, wine, liquor"	Coded as 1 if any increase was reported	M2, Q1, FQ	79 days

© 2023 Scoglio AAJ et al. *JAMA Network Open.*

Use of substances to cope with stress	One item from the Brief COPE scale, "In the past 7 days, I've been using alcohol or other drugs to help me get through it."	Any endorsement: "I've been doing this a little bit/a medium amount/a lot"	Q1	141 days

Abbreviations. PTSS: Posttraumatic stress symptoms; M2: Month two questionnaire; M3: Month three questionnaire; Q1: Quarter one questionnaire; Q2: Quarter two questionnaire; FQ: Final quarterly questionnaire

References: Kroenke K, Spitzer RL, Williams JB. The Patient Health Questionnaire-2: validity of a two-item depression screener. 10.1097/01.MLR.0000093487.78664.3C. *Med Care*. 2003;41(11):1284-92.

Kroenke K, Spitzer RL, Williams JB, Monahan PO, Lowe B. Anxiety disorders in primary care: prevalence, impairment, comorbidity, and detection. 10.7326/0003-4819-146-5-200703060-00004. *Ann Intern Med*. 2007;146(5):317-25.

[†]Average time between exposure and outcome assessments was determined by taking the average between survey return date of the highest distress measure during follow-up and that of the month one questionnaire for each individual. If there are ties, the first occurrence was considered. The average duration varied by outcome because of assessment frequencies and general trend in distress levels over time.

© 2023 Scoglio AAJ et al. *JAMA Network Open.*

eFigure 1. Timeline of Administration of Study Measures.

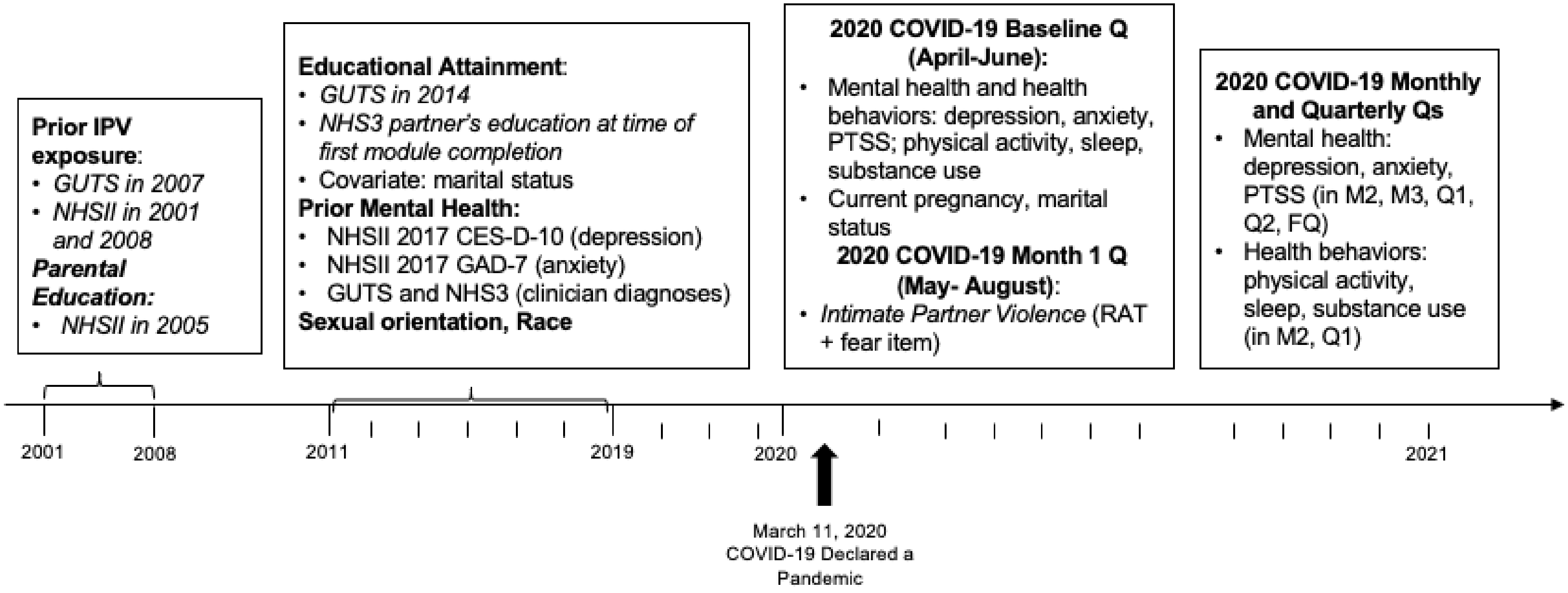

Abbreviations. NHSII: Nurses' Health Study II; GUTS: Grow Up Today Study; NHS3: Nurses' Health Study 3; M2: Month two questionnaire; M3: Month three questionnaire; Q1: Quarter one questionnaire; Q2: Quarter two questionnaire; FQ: Final quarterly questionnaire

© 2023 Scoglio AAJ et al. *JAMA Network Open.*

eTable 2. Associations between the Relationship Assessment Tool score at month one and mental health & health-related behavior during the pandemic.

	Odds Ratio	CI Lower	CI Upper	P-value	FDR P-value
NHSII (N=3503)					
Depression (PHQ4)	1.45	1.34	1.57	6.1E-19	4.9E-18
Anxiety (PHQ4)	1.33	1.24	1.43	3.2E-14	1.9E-13
PTSS (IES-6)	1.27	1.18	1.37	3.6E-10	1.1E-09
Decreased physical activity	1.17	1.09	1.26	2.03E-05	4.4E-05
Poor sleep quality	1.17	1.09	1.27	3.7E-05	7.3E-05
Decreased sleep duration	1.18	1.10	1.28	1.3E-05	3.2E-05
Increased alcohol use	1.11	1.03	1.20	7.3E-03	8.8E-03
Use of alcohol or drugs to cope	1.16	1.06	1.26	7.7E-04	1.2E-03
GUTS (N=2858)					
Depression (PHQ4)	1.41	1.29	1.54	6.8E-14	3.0E-13
Anxiety (PHQ4)	1.23	1.12	1.36	4.7E-05	8.6E-05
PTSS (IES-6)	1.14	1.04	1.24	3.97E-03	5.0E-03
Decreased physical activity	1.03	0.95	1.12	5.2E-01	5.2E-01
Poor sleep quality	1.24	1.14	1.35	9.5E-07	2.5E-06
Decreased sleep duration	1.15	1.06	1.25	1.2E-03	1.6E-03
Increased alcohol use	1.11	1.02	1.21	1.1E-02	1.3E-02
Use of alcohol or drugs to cope	1.12	1.02	1.24	1.7E-02	1.9E-02
NHS3 (N=7236)					
Depression (PHQ4)	1.44	1.36	1.53	4.0E-37	1.0E-35
Anxiety (PHQ4)	1.32	1.25	1.40	1.1E-22	1.3E-21
PTSS (IES-6)	1.23	1.17	1.30	7.2E-14	2.9E-13
Decreased physical activity	1.04	0.99	1.10	1.3E-01	1.3E-01
Poor sleep quality	1.22	1.16	1.29	8.8E-13	3.0E-12
Decreased sleep duration	1.09	1.04	1.15	8.2E-04	1.2E-03
Increased alcohol use	1.09	1.04	1.15	6.4E-04	1.0E-03
Use of alcohol or drugs to cope	1.12	1.05	1.18	2.4E-04	4.3E-04
Meta-analysis (all cohorts)					
Depression (PHQ4)	1.44	1.38	1.50	2.8E-67	2.3E-66
Anxiety (PHQ4)	1.31	1.26	1.36	1.1E-38	4.5E-38
PTSS (IES-6)	1.22	1.15	1.29	5.7E-12	1.1E-11
Decreased physical activity	1.08	0.99	1.17	0.1	7.0E-02
Poor sleep quality	1.21	1.16	1.26	1.1E-21	3.0E-21
Decreased sleep duration	1.13	1.08	1.19	2.2E-06	2.6E-06
Increased alcohol use	1.10	1.06	1.14	5.4E-07	7.2E-07
Use of alcohol or drugs to cope	1.13	1.08	1.18	4.4E-08	7.0E-08

Abbreviations. NHSII: Nurses' Health Study II; GUTS: Grow Up Today Study; NHS3: Nurses' Health Study 3; CI: confidence interval; FDR: false discovery rate.

© 2023 Scoglio AAJ et al. *JAMA Network Open.*

eTable 3. Associations between reported feeling afraid of spouse/partner/significant other at month one and mental health & health-related behavior during the pandemic.

	Odds Ratio	CI Lower	CI Upper	P-value	FDR P-value
NHSII (N=3503)					
Depression (PHQ4)	3.57	1.91	6.69	6.9E-05	8.3E-04
Anxiety (PHQ4)	3.50	1.85	6.59	1.1E-04	8.9E-04
PTSS (IES-6)	2.51	1.33	4.72	4.4E-03	1.5E-02
Decreased physical activity	1.00	0.56	1.78	9.9E-01	9.9E-01
Poor sleep quality	1.50	0.84	2.70	1.7E-01	2.7E-01
Decreased sleep duration	1.59	0.88	2.88	1.3E-01	2.6E-01
Increased alcohol use	1.48	0.82	2.67	1.9E-01	2.7E-01
Use of alcohol or drugs to cope	1.38	0.73	2.61	3.2E-01	3.6E-01
GUTS (N=2858)					
Depression (PHQ4)	3.13	1.72	5.70	1.9E-01	1.1E-03
Anxiety (PHQ4)	1.64	0.80	3.35	1.8E-01	2.7E-01
PTSS (IES-6)	1.52	0.79	2.92	2.1E-01	2.8E-01
Decreased physical activity	1.17	0.65	2.10	6.1E-01	6.7E-01
Poor sleep quality	1.84	1.00	3.37	4.8E-02	1.2E-01
Decreased sleep duration	1.98	1.10	3.58	2.4E-02	7.1E-02
Increased alcohol use	1.41	0.72	2.52	2.4E-01	2.9E-01
Use of alcohol or drugs to cope	1.67	0.84	3.41	1.6E-01	2.7E-01
NHS3 (N=7236)					
Depression (PHQ4)	2.57	1.77	3.72	6.3E-07	1.5E-05
Anxiety (PHQ4)	1.80	1.22	2.64	3.1E-03	1.2E-02
PTSS (IES-6)	1.34	0.92	1.96	1.2E-01	2.6E-01
Decreased physical activity	0.97	0.67	1.39	8.7E-01	9.0E-01
Poor sleep quality	1.82	1.26	2.63	1.4E-01	6.7E-03
Decreased sleep duration	1.30	0.91	1.86	1.5E-01	2.7E-01
Increased alcohol use	1.24	0.88	1.75	2.3E-01	2.9E-01
Use of alcohol or drugs to cope	1.58	1.04	2.41	3.2E-02	8.6E-02
Meta-analysis (all cohorts)					
Depression (PHQ4)	2.87	2.16	3.80	2.2E-13	1.8E-12
Anxiety (PHQ4)	2.12	1.38	3.26	5.8E-04	1.6E-03
PTSS (IES-6)	1.62	1.13	2.34	9.2E-03	1.2E-02
Decreased physical activity	1.01	0.77	1.33	9.2E-01	9.2E-01
Poor sleep quality	1.75	1.33	2.31	7.6E-05	3.0E-04
Decreased sleep duration	1.48	1.13	1.94	4.7E-03	9.4E-03
Increased alcohol use	1.32	1.01	1.72	4.1E-02	4.6E-02
Use of alcohol or drugs to cope	1.55	1.13	2.12	6.5E-03	1.0E-02

Abbreviations. NHSII: Nurses' Health Study II; GUTS: Grow Up Today Study; NHS3: Nurses' Health Study 3; CI: confidence interval; FDR: false discovery rate.

© 2023 Scoglio AAJ et al. *JAMA Network Open.*

eTable 4. Minimally adjusted (age and race/ethnicity adjusted) associations between the Relationship Assessment Tool score at month one and mental health & health-related behavior during the pandemic.

	Odds Ratio	CI Lower	CI Upper	P-value	FDR P-value
NHSII (N=3503)					
Depression (PHQ4)	1.50	1.40	1.62	5.6E-26	4.5E-25
Anxiety (PHQ4)	1.37	1.28	1.47	1.6E-18	7.8E-18
PTSS (IES-6)	1.32	1.23	1.41	3.2E-14	9.7E-14
Decreased physical activity	1.19	1.11	1.28	1.3E-06	2.4E-06
Poor sleep quality	1.21	1.13	1.30	2.0E-07	4.8E-07
Decreased sleep duration	1.21	1.13	1.31	4.3E-07	8.7E-07
Increased alcohol use	1.12	1.04	1.20	2.8E-03	3.4E-03
Use of alcohol or drugs to cope	1.18	1.08	1.28	1.2E-04	1.7E-04
GUTS (N=2858)					
Depression (PHQ4)	1.45	1.33	1.58	2.5E-17	8.5E-17
Anxiety (PHQ4)	1.28	1.16	1.40	3.0E-07	6.5E-07
PTSS (IES-6)	1.18	1.08	1.28	1.4E-04	1.9E-04
Decreased physical activity	1.03	0.95	1.11	5.3E-01	5.3E-01
Poor sleep quality	1.26	1.16	1.37	8.9E-08	2.4E-07
Decreased sleep duration	1.15	1.06	1.25	7.1E-04	9.0E-04
Increased alcohol use	1.13	1.04	1.22	4.1E-03	4.6E-03
Use of alcohol or drugs to cope	1.14	1.04	1.25	5.8E-03	6.3E-03
NHS3 (N=7236)					
Depression (PHQ4)	1.50	1.42	1.59	3.7E-49	8.9E-48
Anxiety (PHQ4)	1.39	1.32	1.46	1.9E-33	2.3E-32
PTSS (IES-6)	1.30	1.23	1.37	3.7E-22	2.2E-21
Decreased physical activity	1.05	1.00	1.10	5.4E-02	5.7E-02
Poor sleep quality	1.27	1.20	1.33	2.9E-18	1.2E-17
Decreased sleep duration	1.12	1.07	1.18	5.9E-06	9.4E-06
Increased alcohol use	1.12	1.06	1.17	1.6E-05	2.5E-05
Use of alcohol or drugs to cope	1.14	1.08	1.21	4.9E-06	8.4E-06
Meta-analysis (all cohorts)					
Depression (PHQ4)	1.49	1.44	1.55	3.1E-90	2.5E-89
Anxiety (PHQ4)	1.36	1.31	1.42	7.0E-56	2.8E-55
PTSS (IES-6)	1.27	1.19	1.35	9.2E-14	1.8E-13
Decreased physical activity	1.09	0.99	1.19	6.8E-02	6.8E-02
Poor sleep quality	1.25	1.20	1.30	2.1E-30	5.5E-30
Decreased sleep duration	1.16	1.10	1.21	5.8E-10	7.7E-10
Increased alcohol use	1.12	1.08	1.16	2.2E-09	2.5E-09
Use of alcohol or drugs to cope	1.15	1.10	1.20	5.5E-11	8.8E-11

Abbreviations. NHSII: Nurses' Health Study II; GUTS: Grow Up Today Study; NHS3: Nurses' Health Study 3; CI: confidence interval; FDR: false discovery rate.

© 2023 Scoglio AAJ et al. *JAMA Network Open.*

eTable 5. Minimally adjusted (age and race/ethnicity adjusted) associations between reported feeling afraid of spouse/partner/significant other at month one and mental health & health-related behavior during the pandemic.

	Odds Ratio	CI Lower	CI Upper	P-value	FDR P-value
NHSII (N=3503)					
Depression (PHQ4)	4.85	2.79	8.43	2.5E-08	3.0E-07
Anxiety (PHQ4)	4.57	2.46	8.48	1.5E-06	1.2E-05
PTSS (IES-6)	3.33	1.86	5.97	5.0E-05	2.2E-04
Decreased physical activity	1.12	0.62	2.01	7.1E-01	7.4E-01
Poor sleep quality	1.79	1.02	3.17	4.4E-02	8.2E-02
Decreased sleep duration	1.81	1.02	3.23	4.3E-02	8.2E-02
Increased alcohol use	1.70	0.95	3.03	7.3E-02	9.7E-02
Use of alcohol or drugs to cope	1.55	0.81	2.95	1.8E-01	2.1E-01
GUTS (N=2858)					
Depression (PHQ4)	3.45	1.89	6.29	5.4E-05	2.2E-04
Anxiety (PHQ4)	1.89	0.98	3.65	5.7E-02	9.1E-02
PTSS (IES-6)	1.69	0.92	3.13	9.3E-02	1.2E-01
Decreased physical activity	1.19	0.66	2.14	5.7E-01	6.2E-01
Poor sleep quality	2.03	1.11	3.68	2.1E-02	4.9E-02
Decreased sleep duration	1.98	1.10	3.56	2.2E-02	4.9E-02
Increased alcohol use	1.49	0.83	2.65	1.8E-01	2.1E-01
Use of alcohol or drugs to cope	1.96	0.99	3.88	5.5E-02	9.1E-02
NHS3 (N=7236)					
Depression (PHQ4)	2.97	2.13	4.14	1.7E-10	4.1E-09
Anxiety (PHQ4)	2.14	1.51	3.05	2.4E-05	1.4E-04
PTSS (IES-6)	1.64	1.16	2.30	4.6E-03	1.4E-02
Decreased physical activity	1.04	0.73	1.48	8.3E-01	8.3E-01
Poor sleep quality	2.00	1.41	2.84	1.2E-04	4.0E-04
Decreased sleep duration	1.40	0.98	1.99	6.2E-02	9.3E-02
Increased alcohol use	1.37	0.97	1.93	7.0E-02	9.7E-02
Use of alcohol or drugs to cope	1.67	1.11	2.51	1.4E-02	3.6E-02
Meta-analysis (all cohorts)					
Depression (PHQ4)	3.46	2.57	4.65	2.1E-16	1.7E-15
Anxiety (PHQ4)	2.59	1.57	4.26	1.9E-04	5.0E-04
PTSS (IES-6)	2.03	1.31	3.13	1.4E-03	1.9E-03
Decreased physical activity	1.09	0.83	1.42	5.5E-01	5.5E-01
Poor sleep quality	1.96	1.50	2.56	8.7E-07	3.5E-06
Decreased sleep duration	1.59	1.22	2.08	6.8E-04	1.2E-03
Increased alcohol use	1.46	1.12	1.89	4.9E-03	5.6E-03
Use of alcohol or drugs to cope	1.69	1.25	2.30	7.7E-04	1.2E-03

Abbreviations. NHSII: Nurses' Health Study II; GUTS: Grow Up Today Study; NHS3: Nurses' Health Study 3; CI: confidence interval; FDR: false discovery rate.

© 2023 Scoglio AAJ et al. *JAMA Network Open.*

eFigure 2. Prevalence of intimate partner violence assessment items in the analytic samples across three cohorts.

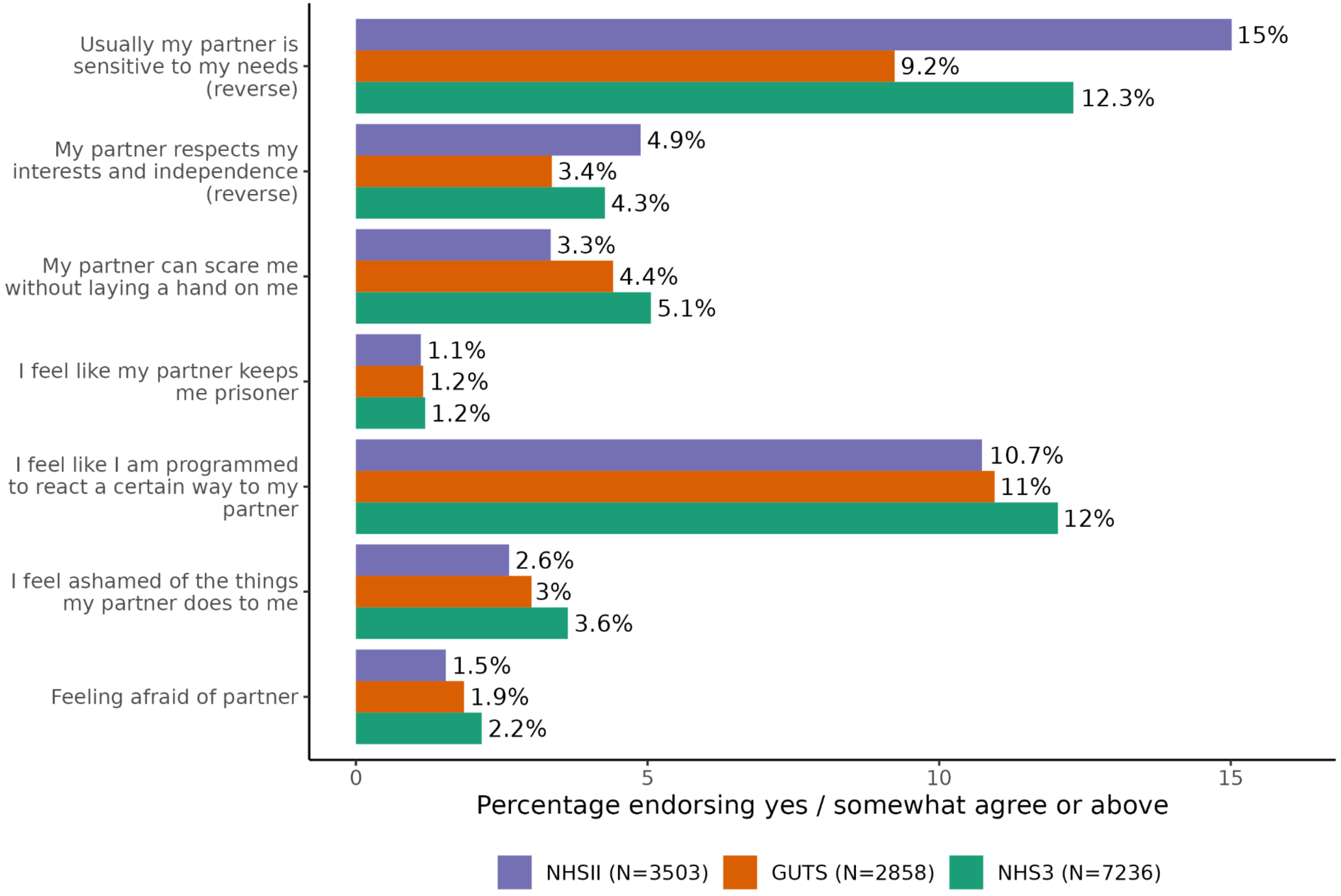

Abbreviations. NHSII: Nurses' Health Study II; GUTS: Grow Up Today Study; NHS3: Nurses' Health Study 3

© 2023 Scoglio AAJ et al. *JAMA Network Open.*

© 2023 Scoglio AAJ et al. *JAMA Network Open.*
