# Supplementary material for: Intimate Partner Violence, Mental Health Symptoms, and Modifiable Health Factors in Women During the COVID-19 Pandemic in the US
Source: JAMA Netw Open. 2023 Mar 14;6(3):e232977. doi: 10.1001/jamanetworkopen.2023.2977 (PMC10015312; doi:10.1001/jamanetworkopen.2023.2977)
Supplement: Supplement 2. — Data Sharing Statement [file jamanetwopen-e232977-s002.pdf]

Data Sharing Statement

Scoglio. Intimate Partner Violence, Mental Health Symptoms, and Modifiable Health Factors in Women During the COVID-19 Pandemic in the US. *JAMA Netw Open*. Published March 14, 2023. doi:10.1001/jamanetworkopen.2023.2977

Data
Data available: No

Additional Information
Explanation for why data not available: Authors do not have permission to share data.
